# METTL13 is essential for the survival of acute myeloid leukemia cells by regulating MYC

**DOI:** 10.1038/s41420-025-02512-x

**Published:** 2025-05-17

**Authors:** Kui Zhao, Hanyue Zhang, Shuoting Wang, Yuhang Zhou, Zhishuai Zhang, Baoqiang Kang, Huaisong Lin, Yanqi Zhang, Jiaming Gu, Carla Pantoja, Lingling Liu, Yi He, Guangjin Pan, Yongli Shan, Bing Long

**Affiliations:** 1 Department of Hematology, The Third Affiliated Hospital, Sun Yat-sen University, 510630 Guangzhou, China; 2 Department of Gastroenterology, The Eighth Affiliated Hospital, Sun Yat-sen University, 518033 Shenzhen, China; 3 Guangdong Provincial Key Laboratory of Stem Cell and Regenerative Medicine, Guangzhou Institutes of Biomedicine and Health, Chinese Academy of Sciences, 510530 Guangzhou, China; 4 Department of Cell Biology, Yale University, New Haven, CT USA

**Keywords:** Acute myeloid leukaemia, Acute myeloid leukaemia

## Abstract

Recently, some methyltransferase-like (METTL) proteins have been found to play crucial roles in the development of acute myeloid leukemia (AML) through mediating RNA modifications, such as METTL3/14/16 mediated N^6^-methyladenosine (m^6^A) and METTL1 mediated N^7^-methylguanosine (m^7^G). However, the roles of other METTL proteins in AML progression remain unknown. Here, we examined the expression levels of all METTL members in AML samples and showed that METTL13 was increased in AML and positively correlated with poor prognosis. Moreover, METTL13 deficiency impaired AML cell proliferation capability in vitro, improved the survival of AML cell line xenograft immune-deficient mice, and reduced tumor infiltration in vivo. Mechanistically, MYC was downregulated after METTL13 knockdown and forced expression of MYC rescued the cell proliferation defect in METTL13-deficient AML cells. Our findings uncover the critical role of METTL13 in the survival of AML cells and identify MYC as a potential downstream target of METTL13. This work highlights METTL13 as a promising candidate target for AML therapy.

## Introduction

Acute myeloid leukemia (AML) is the most common heterogeneous hematologic malignancy with a 5-year survival of only 28.3% in adults, exhibits poor prognosis under primary chemoresistance, and a high relapse rate in patients because of the diverse triggering factors and complex microenvironment [1, 2]. The future of AML therapy lies in combining complementary immunotherapies with specific chemotherapeutics or inhibitors targeting other oncogenic pathways [3]. Thus, identifying novel, reliable, and specific biomarkers in AML pathogenesis is crucial. Epigenetic regulation, such as DNA methylation and post-translational histone modifications, plays an important role in embryonic development and many diseases, including AML pathogenesis [4, 5]. Drugs targeting epigenetic enzymes which mediate these modifications have already been applied in clinical practice [6].

Recently, RNA methylation, such as N^6^-methyladenosine (m^6^A) and N^7^-methylguanosine (m^7^G), catalyzed by methyltransferase-like proteins (METTL) have been shown to be involved in hematopoiesis and malignancies [7]. For example, m^6^A mediated by METTL3 and METTL14 methyltransferase complex is an abundant RNA modification on mammalian mRNA [8, 9], which plays important roles in hematopoietic development and the maintenance of hematopoietic and leukemia stem cells [10–14]. Especially, METTL3 and METTL14 are also involved in various aspects of the occurrence and development of AML dependent or independent on their m^6^A methyltransferase activity [7], including maintenance of the leukemic state [15], therapeutic resistance [16], and leukemia cell survival and differentiation [10, 17]. Another m^6^A methyltransferase, METTL16, has also been confirmed to drive tumorigenesis and leukemogenesis and maintain leukemia stem cell self-renewal [18]. Lastly, METTL1 and its mediating of m^7^G modification on tRNA have been reported to promote cell proliferation and induce apoptosis in AML cells [19–22]. These data indicate that multiple members of the METTL family and their catalytic RNA modifications are involved in AML progression. However, the precise roles and mechanisms of other METTL members in AML progression remain unclear.

In this work, to illustrate the roles of other METTL members in AML, we first examined the expression level of all 34 METTL members in AML and normal samples, and found METTL13 was upregulated in AML samples and associated with poor prognosis. Recently, two independent research groups have demonstrated that METTL13 can enhance RNA translation in hepatocellular carcinoma and pancreatic cancer cells through mediating eEF1A methylation [23, 24]. However, the role of METTL13 in AML has not been reported. In this study, we knocked down METTL13 in AML cells and found that METTL13 deficiency impaired AML cell proliferation and survival through downregulation of MYC expression. Our research reveals the essential roles of METTL13 in AML survival by regulating MYC and provides a potential target candidate for AML therapy.

## Results

### METTL13 is positively associated with AML and poor prognosis

**Fig. 1:**
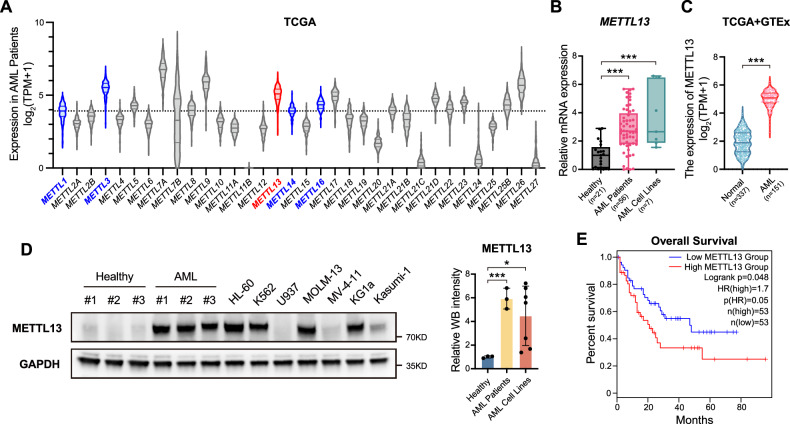
METTL13 was upregulated and associated with poor prognosis of AML. **A** Expression level of METTL family in AML patients from TCGA database. The members confirmed to promote AML progression in other research were marked in blue (METTL1/3/14/16), while the focus in this study, METTL13, was marked in red. **B** Expression of METTL13 in healthy individuals, clinical AML patients and cell lines. **C** Expression of METTL13 in AML and non-matched normal samples in the TCGA and GTEx databases. **D** Western blotting using normal bone marrow of healthy individuals and AML patients, and cell lines (HL-60, K562, U937, MOLM-13, MV-4-11, KG1a and Kasumi-1). Fluorescence intensity was quantified by Fiji software. **E** Correlation between METTL13 and overall survival of AML patients from GEPIA database (http://gepia.cancer-pku.cn). Data were presented as mean ± SD (Student’s test, *p < 0.05, ***p < 0.001).

To resolve the raised questions regarding the roles of all METTL members in AML progression, we first assessed the expression levels of 34 members of the METTL proteins. Based on The Cancer Genome Atlas Program (TCGA) database, we collected the expression profiles of 151 AML patients. Because METTL1, METTL3, METTL14, and METTL16 were significantly associated with AML development in the previous reports, we set METTL1 as the baseline due to its lowest expression level among these four genes, and focused on the METTL proteins above the expression level of METTL1. Interestingly, some members, such as METTL7A, METTL9, METTL13, METTL18, and METTL26, exhibited high expression levels according to the TCGA database (Fig. 1A). Consistently, we performed RT-qPCR assays to detect the mRNA levels of 34 METTL members in healthy cells, AML primary cells, and cell lines and confirmed the increased level of METTL13 in AML samples (Fig. S1). Indeed, METTL13 was upregulated in AML at the mRNA level, as evidenced by RT-qPCR assays with more clinical samples (Fig. 1B). Furthermore, a combined analysis of AML samples from the TCGA database and normal samples from the Genotype-Tissue Expression (GTEx) database confirmed METTL13 upregulation in AML (Fig. 1C). This finding was further supported by protein overexpression demonstrated by Western blot analysis (Fig. 1D). These data suggest that METTL13 is positively associated with AML. To validate the relationship between METTL13 and AML prognosis, we conducted a survival analysis using the gene expression profiling interactive analysis (GEPIA) database and showed that high expression of METTL13 was associated with poor survival (Fig. 1E). Together, these data suggest that METTL13 might play a critical role in AML development.

### METTL13 is required for the proliferation and survival of AML cells

**Fig. 2:**
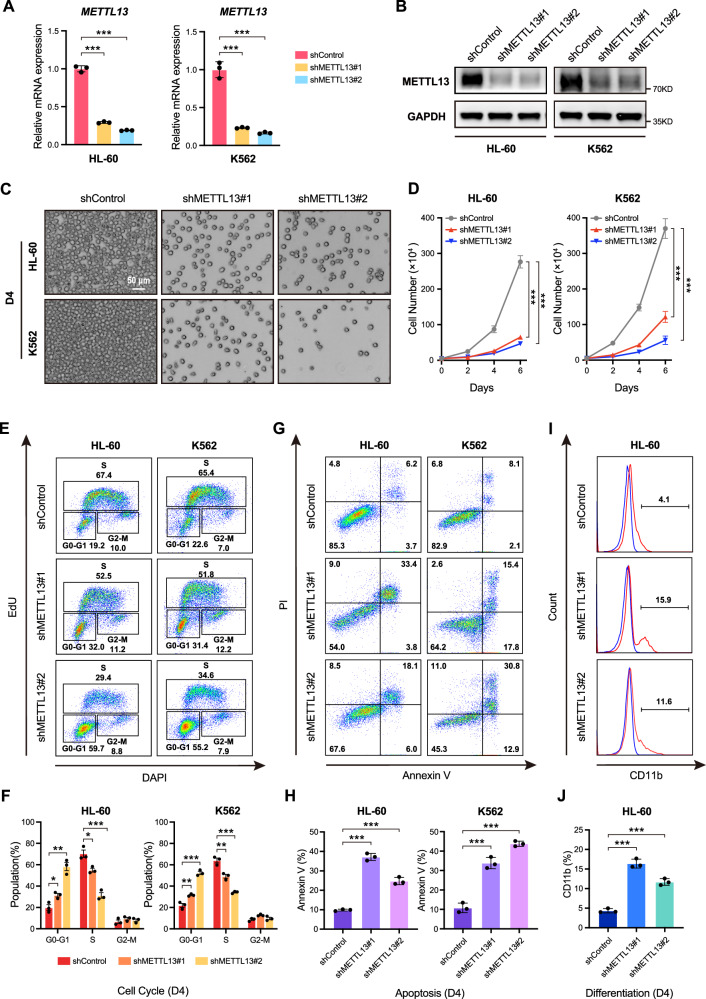
METTL13 knockdown inhibited the survival and growth of human AML cells. **A** and **B** Validation of the knockdown effect of METTL13 using RT-qPCR and Western blot in HL-60 and K562 cells. **C** Representative growth images of HL-60 and K562 cells on day 4 after METTL13 knockdown. **D** Proliferation trend of HL-60 and K562 cells by cell counting after METTL13 knockdown. **E** and **F** Cell cycle distribution of HL-60 and K562 cells conducted by flow cytometry on day 4 after METTL13 knockdown. **G** and **H** Percentages of apoptotic HL-60 and K562 cells were conducted by flow cytometry on day 4 after METTL13 knockdown. **I** and **J** Expression analysis of CD11b in HL-60 cells on day 4 after METTL13 knockdown. Data were presented as mean ± SD (Student’s test, *p < 0.05, **p < 0.01, ***p < 0.001).

**Fig. 3:**
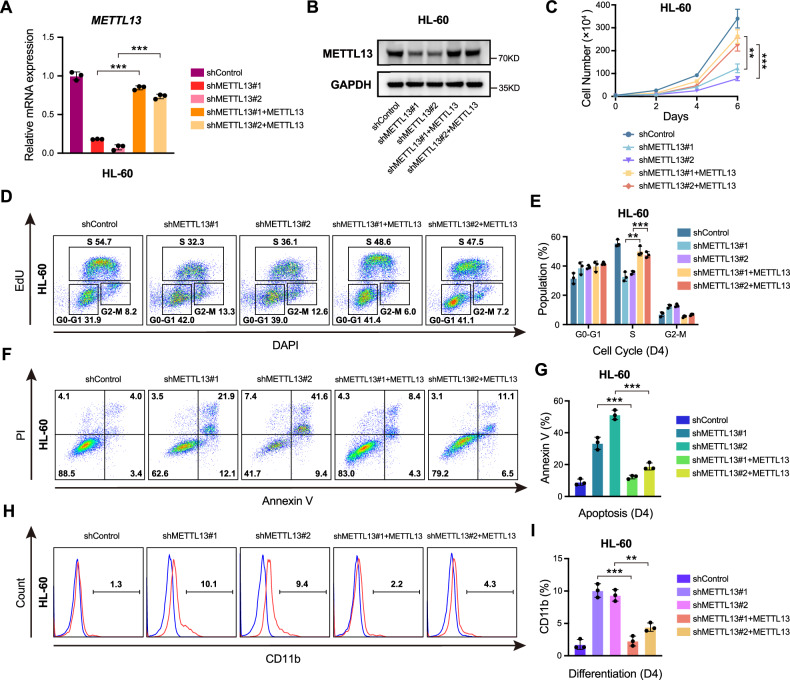
Re-expression of METTL13 rescued the proliferation defect induced by METTL13 knockdown in HL-60 cells. **A** and **B** Validation of the exogenous expression effect of METTL13 after METTL13 knockdown by RT-qPCR and western blot in HL-60 cells. **C–I** Ectopic expression of METTL13 rescued the proliferation of METTL13-knockdown HL-60 cells: **C** growth curves, **D**, **E** cell cycle, **F**, **G** apoptosis, and **H**, **I** differentiation.

To further explore the functional roles of METTL13 in AML progression, we knocked down METTL13 (METTL13-KD) using lentivirus expressing short hairpin RNA (shRNA) targeting *METTL13* (sh*METTL13*#1 and sh*METTL13*#2) in HL-60 and K562 cell lines (Fig. 2A and B). Excitingly, METTL13 deficiency resulted in poor proliferation capability of the leukemia cell lines as assessed by representative growth images and cell number counting (Fig. 2C and D). Similarly, the proliferation of primary cells and other AML cell lines was inhibited (Fig. S2A–C). Consistently, METTL13-KD showed substantially reduced EdU incorporation and decreased S-phase of the cell cycle in HL-60 and K562 lines, while the G1 phase in the cell cycle was increased (Fig. 2E and F). This indicates that METTL13-KD in AML cells leads to cell cycle arrest. Notably, more apoptosis was detected in METTL13-KD HL-60 and K562 cells than in control cells (Fig. 2G and H). On the other hand, AML differentiation could affect the proliferation capability of AML [25]. Indeed, METTL13-KD increased the expression of the differentiation marker CD11b in HL-60 cells (Fig. 2I and J), suggesting that METTL13 represses the differentiation potential of AML cells. We further showed that the proliferation and survival defects in METTL13-KD cells could be rescued by the re-expression of exogenous METTL13 in METTL13-KD HL-60 cells (Fig. 3A–I), suggesting the phenotype is specific to METTL13 in AML. Together, these data demonstrate that METTL13 is required for the proliferation and survival of AML cells.

### METTL13 is essential for the survival of AML cells in vivo

**Fig. 4:**
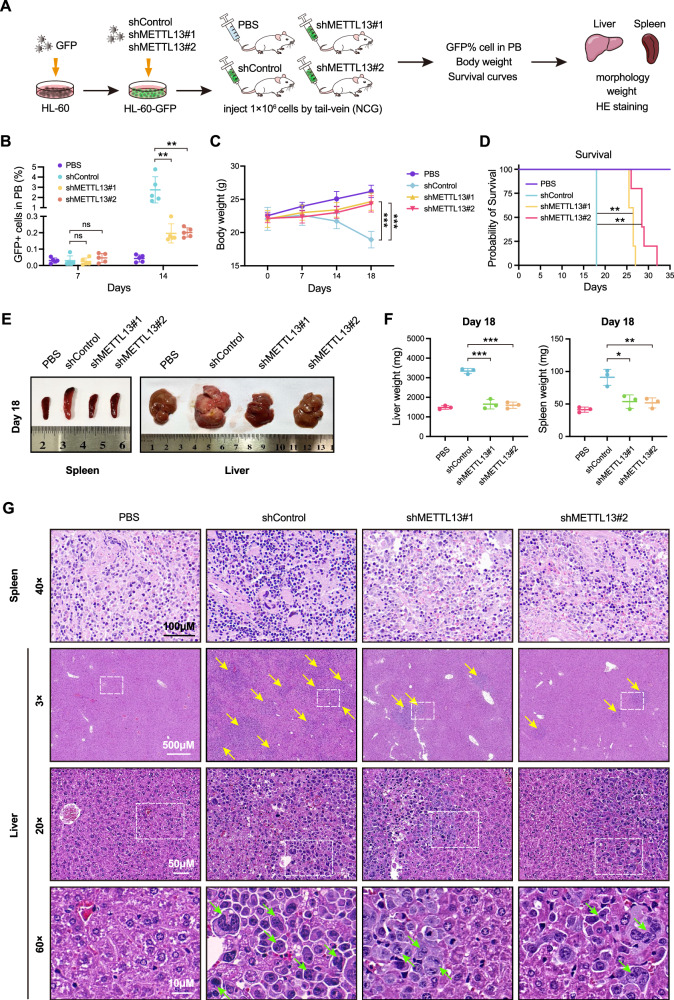
METTL13 knockdown suppressed the survival of AML cells in vivo. **A** Schematic diagram of in vivo experimental design. **B** GFP^+^ leukemic cells in peripheral blood were analyzed using flow cytometry as indicated. **C** The trend in body weight variation across the four groups of mice. **D** Kaplan–Meier survival curves of four groups of mice. Differences were compared by the log-rank test. **E** and **F** Size and weight of spleen and liver of four groups of mice. **G** Representative images of tumor cell infiltration in spleen and liver using H&E staining. The yellow arrows pointed to the tumor lesion. The white dashed box outlined the further magnified field of view. The green arrows point to the tumor cells exhibiting pronounced atypia. Data were presented as mean ± SD (Student’s test, *p < 0.05, **p < 0.01, ***p < 0.001).

To further investigate the essential roles of METTL13 in the survival of AML in vivo, we generated xenograft mice models of AML and performed a series of animal experiments (Fig. 4A). First, we labeled HL-60 cells with lentivirus-expressing GFP (HL-60-GFP) to easily track the transplanted cells for further detection, and then knocked down METTL13 in HL60-GFP cells (METTL13-KD HL-60-GFP). Next, 1 × 10^6^ METTL13-KD cells or control cells were transplanted via tail-vein injection into 6-week-old female NOD/ShiLtJGpt-Prkdc^em26Cd52^Il2rg^em26Cd22^/Gpt (NCG) mice. AML progression was comprehensively assessed by monitoring the population of GFP^+^ cells in peripheral blood (PB), mouse body weight, survival time, and histological staining of the liver and spleen (Fig. 4A). Interestingly, mice inoculated with METTL13-KD HL-60-GFP cells showed a significantly lower number of GFP^+^ cells in their peripheral blood than that of the control group (Fig. 4B). Consistent with our above results in vitro, METTL13-KD mice showed a slower rate of weight loss (Fig. 4C) and prolonged survival time compared to the control group (Fig. 4D), suggesting that METTL13 promotes the survival of AML cells in vivo. After 18 days post-transplantation, the mice inoculated with METTL13-KD HL-60-GFP cells had significantly smaller sizes and weights of spleen and liver than that of the control group (Fig. 4E and F). Moreover, there was less tumor cell infiltration in the spleen and liver in METLL13-KD AML mice (Fig. 4G). Together, these results demonstrate that METTL13 is essential for the proliferation and survival of AML cells in vivo.

### Transcriptome alterations in AML cells resulting from METTL13 knockdown

**Fig. 5:**
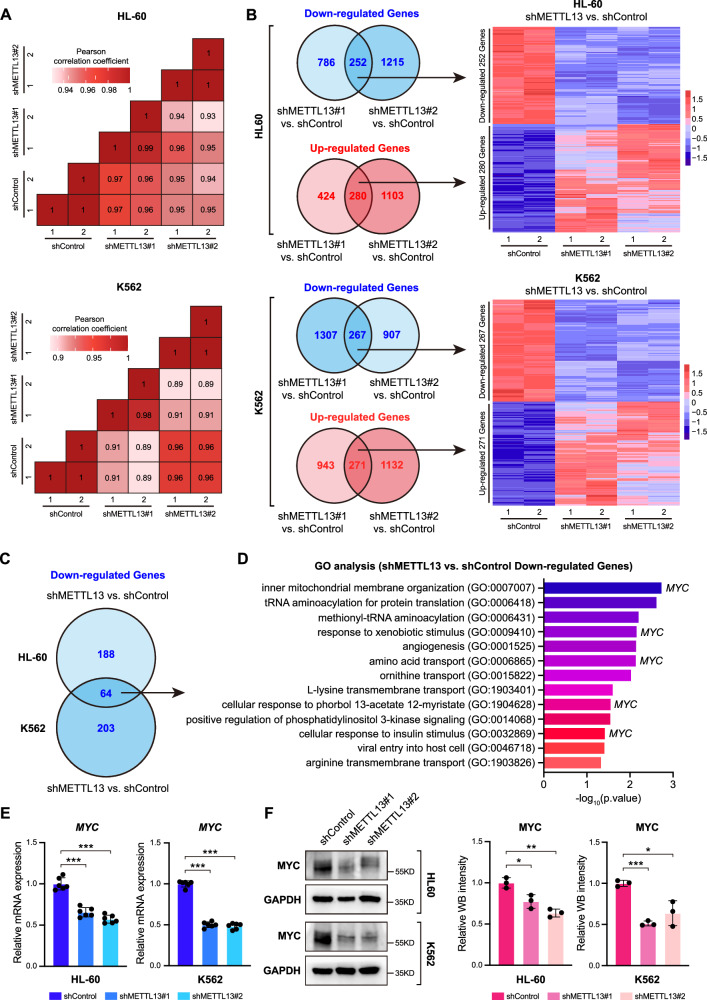
Transcriptomic alterations in leukemia cells after METTL13 knockdown. **A** Pearson correlation analysis on the whole-genome transcriptome of HL-60 and K562 cells after METTL13 knockdown. **B** Venn diagrams illustrate the number of genes co-upregulated or co-downregulated after METTL13 knockdown using two different shRNA in HL-60 and K562 cells, respectively (left panel). These genes were presented in heatmaps (right panel). **C** The Venn diagram illustrates genes co-downregulated in HL-60 and K562 cells after METTL13 knockdown. **D** GO analysis (BP) of signaling pathways enriched in co-downregulated genes in panel **C**. **E** and **F** Validation of the expression level of MYC by RT-qPCR and western blot in HL-60 and K562 cells. Data were presented as mean ± SD (Student’s test, *p < 0.05, **p < 0.01, ***p < 0.001).

To decipher the mechanisms underlying the AML proliferation defect due to the absence of METTL13, we analyzed whole genomic transcriptome profiles in METTL13-KD and control HL-60 and K562 cells by RNA sequencing (RNA-seq). Indeed, METTL13-KD induced whole-transcriptome changes in HL-60 and K562 cells compared to control group as assessed by Pearson correlation analysis (Fig. 5A). Because there were two shRNAs targeting METTL13, we analyzed shared differentially expressed genes upon treatment of the two shRNAs in AML cells and showed that METTL13-KD resulted in 252 downregulated genes and 280 upregulated genes in HL-60 cells, and 267 downregulated genes and 271 upregulated genes in K562 cells compared to their corresponding control groups (Fig. 5B). To confirm the potential target genes of METTL13, we further identified the 64 commonly downregulated genes in both HL-60 and K562 cells in the absence of METTL13 (Fig. 5C). These 64 overlapping downregulated genes were related to inner mitochondrial membrane organization, tRNA aminoacylation for protein translation, and methionyl-tRNA aminoacylation, etc. (Fig. 5D). Interestingly, the proto-oncogene MYC is involved in the majority of these pathways (Fig. 5D). Indeed, the mRNA and protein levels of MYC were downregulated in METTL13-KD HL-60 and K562 cells as evidenced by RT-qPCR and Western blot results (Fig. 5E and F). Together, these data suggest that METTL13 knockdown leads to whole-genomic transcriptome changes in AML cells by decreasing the expression level of MYC.

### MYC mediates the function of METTL13 in AML cells

**Fig. 6:**
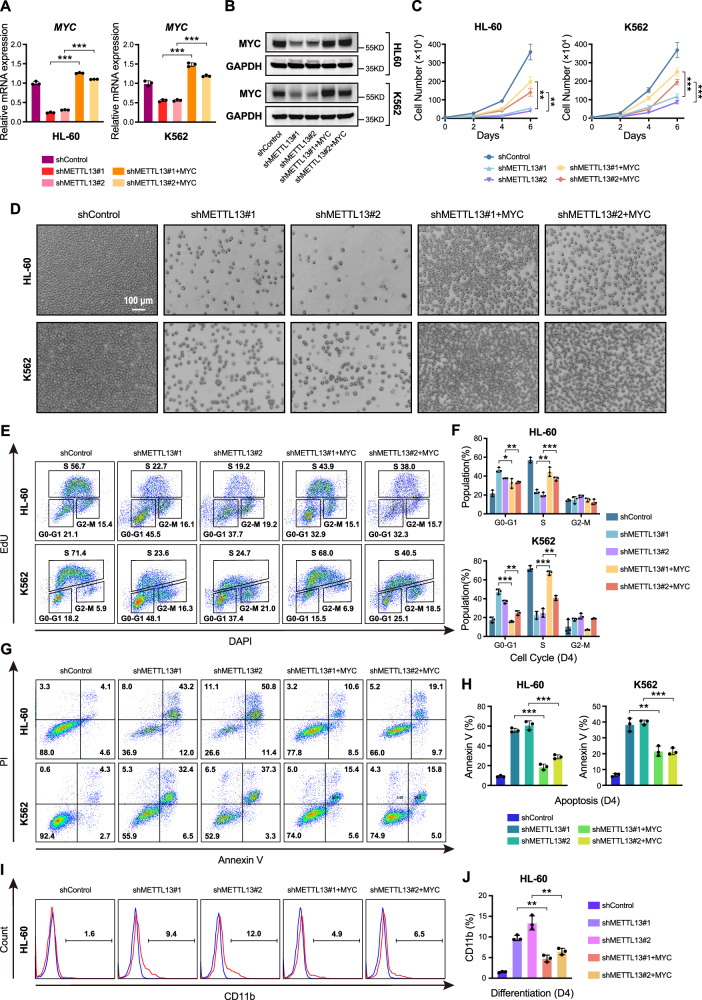
Re-expression of MYC rescued the proliferation defect induced by METTL13 knockdown in AML cells. **A** and **B** Validation of the re-expression level of MYC by RT-qPCR and western blot in HL-60 and K562 cells after METTL13 knockdown. **C** Proliferation curves of HL-60 and K562 cells with METTL13 knockdown followed by MYC re-expression. **D** Representative growth images of HL-60 and K562 cells on day 4 after METTL13 knockdown and MYC re-expression. **E** and **F** Cell cycle distribution of HL-60 and K562 cells conducted by flow cytometry after METTL13 knockdown and MYC re-expression. **G** and **H** Percentages of apoptotic leukemia cells conducted by flow cytometry after METTL13 knockdown and MYC re-expression. **I** and **J** Expression analysis of CD11b in HL-60 cells after METTL13 knockdown and MYC re-expression. Data were presented as mean ± SD (Student’s test, *p < 0.05, **p < 0.01, ***p < 0.001).

Conventionally, MYC plays important roles in cell cycle progression, apoptosis, and cellular transformation [26, 27]. To ascertain whether MYC serves as a potential target gene regulated by METTL13, we expressed exogenous MYC in both METTL13-KD HL-60 and K562 cells (Fig. 6A and B) and found that exogenously expressed MYC restored cell number and proliferation rate (Fig. 6C and D). Importantly, the overexpression of MYC also rescued cell cycle and survival defects in METTL13-KD HL-60 and K562 cells (Fig. 6E–H). Specifically, our results showed overexpressing MYC resulted in an increase in S-phase cells and a decrease in G1-phase cells (Fig. 6E and F). Simultaneously, the overexpression of MYC also inhibits the apoptosis process induced by METTL13-KD (Fig. 6G and H). Moreover, the overexpression of MYC reduced the expression of the myeloid differentiation marker CD11b in METTL13-KD HL-60 and K562 cells (Fig. 6I and J). Taken all the data together, our data indicate that METTL13 regulates the proliferation and survival of AML cells by maintaining the expression level of MYC.

## Discussion

In this study, we delineated the expression levels of 34 METTL members between clinical AML patients and healthy individuals. Conventionally, METTL proteins have been recognized to mainly catalyze various RNA modifications and contribute to epigenetic or epitranscriptomic regulation of gene expression [28]. Several members of this family (METTL1, METTL3, METTL14, and METTL16) have been shown to participate in regulating AML progression [7–22, 29–32]. Here, we showed another METTL member, METTL13, was increased in AML samples. In fact, the upregulation of METTL13 has been detected in many cancers and is associated with worse prognoses, such as breast cancer, head and neck squamous cell carcinoma, nasopharyngeal carcinoma, and gastric cancer [33–36]. In this work, we provided clear evidence to illustrate the essential roles of METTL13 in the proliferation of AML cells in vitro and in vivo. More importantly, we demonstrated that METTL13 is required for the survival of AML cells, suggesting that METTL13 could be a potential target for the diagnosis and personalized precision treatment of AML. Interestingly, METTL13 deficiency had several effects on the proliferation of CD34^+^ cells from healthy donors and markedly suppressed the proliferation of Jurkat cells (Fig. S3). Furthermore, in this work, we focused on METTL13, but the roles of other differential expressed METTL members in AML need to be explored in the future.

METTL13 was identified as a dual methyltransferase that catalyzes methylation of eukaryotic elongation factor 1-alpha (eEF1A1 and eEF1A2) at two different positions [23, 24]. Especially, METTL13 can mediate the methylation of eEF1A at the N terminus and lysine 55 (eEF1AK55me2), and eEF1AK55me2 is increased in pancreatic and lung cancers and associated with poor clinical outcomes [23, 24]. These research studies have proven that METTL13 is involved in the protein translation process. In this work, we found a reduction in various amino acid transport (ornithine, l-lysine, and arginine) and tRNA-related biological processes upon METTL13 loss (Fig. 5D), suggesting that METTL13 may regulate RNA translation to affect the survival of AML cells. Additionally, RNA-seq data from this research revealed that METTL13 depletion alters the transcriptome of AML cells. It remains to determine whether the altered transcriptome (or phenotypes/mechanisms) is dependent or independent of the eEF1AK55me2 pathway in METTL13-KD AML cells. Further experimental studies are required to validate this finding in the future.

The proto-oncogene MYC is a critical effector of tumorigenesis through regulating cell cycle and apoptosis and is aberrantly expressed in approximately 70% of human cancers [26, 27]. Some studies have identified MYC as a downstream target of METTL-mediated epigenetic modifications during the progression of AML [10, 17, 37, 38]. Indeed, MYC was down-regulated upon METTL13 knockdown in AML cells, and overexpressed MYC could rescue the proliferation and survival defects in METTL13-KD AML cells. These results indicate the critical roles of METTL13 in AML survival by regulating MYC. MYC downregulation at the mRNA level may be attributed to decreased transcription or reduced RNA stability, suggesting METTL13 modulates not only translation but also transcription or the post-transcription process to regulate gene expression. On the other hand, RNA modification by METTL13, like other METTL superfamily members, may depend on its RNA recognition and enzymatic activity [39, 40]. For example, another member, METTL3, regulates gene expression co-transcriptionally with other histone modifiers and modifications, such as H3K36me3 and H3K9me2/3, which alter its chromatin binding capability [41, 42]. Hence, METTL13 may directly regulate the transcription, mRNA stability, and translation of MYC in a manner dependent on its DNA/RNA binding capability or enzymatic activity, or indirectly regulate MYC expression dependent on other intermediary molecules. In the future, we will explore the interaction between METTL13 and MYC to clarify how METTL13 regulates MYC expression.

In conclusion, our study found that METTL13 was highly expressed in AML samples, and METTL13 regulated the proliferation and survival of AML cells through modulating MYC expression. Our research uncovers the critical roles of METTL13 in AML survival by regulating MYC and provides a potential target candidate for AML therapy.

## Materials and methods

### Public database analysis

Transcriptome data were downloaded from The Cancer Genome Atlas Program (TCGA, https://cancer.gov/ccg/research/genome-sequencing/tcga) and The Genotype-Tissue Expression (GTEx, https://www.gtexportal.org/) database. Gene expression levels of 34 members from METTL were extracted from AML samples from TCGA. Then, AML samples from TCGA and normal samples from GTEx were combined and expression levels of METTL13 were compared. Overall survival based on METTL13 expression levels was plotted using the Gene Expression Profiling Interactive Analysis (GEPIA, http://gepia.cancer-pku.cn) database.

### Cell culture

Human AML cell lines were grown in RPMI 1640 medium (Gibco, Cat# 1640) with 10% or 20% fetal bovine serum (Vistech, Cat# SE100-011) (HL-60, K562, U937, MOLM-13, MV-4-11, KG1a were 10%, Kasumi-1 was 20%). Primary cells from AML patients were cultured in RPMI 1640 medium with 20% FBS, 1% insulin–transferrin–selenium (Gibco, Cat# 41400045), and GM-CSF (40 ng/mL). Cells were changed with fresh medium every 2–3 days.

### Primary samples from AML patients

Diagnostic bone marrow (BM) aspirates were obtained from AML patients treated at the Third Affiliated Hospital of Sun Yat-sen University. All samples were collected after obtaining informed consent in accordance with the Declaration of Helsinki. The study was approved by the ethics committees of the Institutional Review Boards (IRB protocol approval number: [2022]02-139). Mononuclear cells were derived from BM by Ficoll density gradient centrifugation.

### RNA extraction and RT-qPCR analysis

Total RNA was extracted by RaPure Total RNA Micro Kits (Magen, Cat# R4012-03-250T) and reverse transcribed into cDNA according to the manufacturer’s instructions (Toyobo). RT-qPCR experiment was performed using ChamQ SYBR qPCR Master Mix (Vazyme, Cat# Q311-02) with the CFX-96 Touch machine (Bio-Rad).

### Western blot

RIPA buffer (Beyotime Cat# P0013B) was used to lyse cells on ice. The whole cell extracts treated with sonication were subjected to 4–12% SmartPage Precast Protein Gel Plus (Smart-Lifescience, Cat# SLE020), then transferred to PVDF membranes (Millipore), and incubated with primary antibodies overnight at 4 °C. Next, the membranes were incubated with HRP-conjugated secondary antibody for 2 h at room temperature. After washing three times for 10 min each time, the membranes were detected by ECL (Beyotime, DW111-02) and visualized with a SmartChemi Image Analysis System (Sage Creation). The following antibodies were used: Rabbit anti-METTL13 (Bethyl, Cat# A304-195A), Rabbit anti-MYC (Cell Signaling Technology, Cat# D84C12), HRP-GAPDH (KangChen Bio-tech, Cat# KC-5G5), HRP Goat anti-Rabbit IgG (ABclonal, Cat# AS014). All original Western blot data are provided in the supplementary information.

### Generation and infection of virus

Two different specific shRNA targeting METTL13 for knockdown assay were used (shMETTL13#1: GCCTGTCTTTGCCTTCATCAT, shMETTL13#2: GCTGAAGGATGTGTCTCACAA). These shRNAs were constructed into the pLKO.1 lentivirus vector. Then, these vectors were co-transfected with package vectors into 293 T cells. Next, the generated viruses were collected and used to infect leukemia cells. In the rescue experiment, viruses overexpressing METTL13 or MYC were generated by reconstruction of pSin lenti-virus vector and co-infected with shRNA viruses into leukemia cells.

### Cell counting assay

After validating knockdown or exogenous expression efficacy, 5 × 10^4^ leukemia cells were plated onto six-well plates and counted with Trypan blue staining every two days.

### Cell cycle assay

Cell cycle detection was performed using Click-iT Plus EdU Alexa Fluor 647 Flow Cytometry Assay Kit (Thermo Fisher Scientific, Cat# C10634,) in accordance with the manufacturer’s instruction. 1 × 10^6^ cells were plated onto six-well plates with EdU at 37 °C for 1 h. Next, these cells were fixed in a fixation buffer at room temperature for 15 min. Subsequently, the cells were washed in PBS and permeabilized in perm/wash buffer at 4 °C for 15 min. After washing, the cells were incubated in PBS with CuSO_4_, the fluorescent dye picolyl azide, reaction buffer additive, and DAPI at room temperature for 30 min. Finally, the cells were analyzed by CytoFLEX Flow Cytometer (Beckman).

### Cell apoptosis assay

Apoptosis analysis was performed with Annexin V-FITC/PI Apoptosis Detection Kit (Vazyme, Cat# A211-02) according to the manufacturer’s recommendations. After collecting and washing with PBS, cells were incubated with binding buffer, Annexin V-FITC and PI at room temperature for 15 min in the dark. Then, the cells were analyzed by CytoFLEX Flow Cytometer (Beckman).

### Cell differentiation assay

After washing with PBS, the cells were incubated with CD11b antibody (BioLegend, Cat# 101212) for 30 min at 4 °C. The cells were washed again and resuspended in 200 μL PBS, and then analyzed by CytoFLEX Flow Cytometer (Beckman).

### Xenograft mice model of AML

Human AML HL60 cells were first transduced with a pWPXLd-luciferase-EGFP plasmid by lenti-virus and the GFP + HL-60 cells were purified using flow cytometry. Then gene targets were knocked down by shRNA in these cells and the knockdown efficiency was validated. Forty 6-week-old female NOD/ShiLtJGpt-Prkdc^em26Cd52^Il2rg^em26Cd22^/Gpt (NCG) mice were divided into four groups and transplanted with 100 μL PBS (Mock) or 1 × 10^6^ HL-60 cells (HL-60-shControl, HL-60-shMETTL13#1, HL-60-shMETTL13#2) suspended in 100 μL 1640 medium by tail-vein injection, respectively. Tumor growth was monitored every week through flow cytometry while recording mice's body weight and survival time. Three mice from each of the four groups were randomly selected for dissection at the same time, and HE staining was performed on the spleen and liver to assess tumor infiltration. Animal experiments were conducted in a blind fashion to the investigators. All animal experiments were performed according to protocols approved by the institutional animal care and use committee (IACUC protocol approval number: GIBH-IACUC-2022010).

### RNA-seq and data processing

Total RNA from HL-60-shControl (*n* = 2), HL-60-shMETTL13#1 (*n* = 2) and HL-60-shMETTL13#2 (*n* = 2) cells were isolated with Trizol reagent (MRC, Cat# TR118), then reverse transcribed to create a cDNA library, which was then sequenced (Illumina) by Annoroad Gene Technology Co., Ltd. (Beijing, China). Then, all RNA-Seq data were analyzed. Briefly, reads were aligned to the human genome (UCSC hg38) using HISAT2 (v2.0.4), and gene expression was determined using SAMtools (v1.3.1) and htseq-count (v0.6.0), filtered by a threshold of at least 20 average raw read counts among samples, and then normalized by GC content and gene length using EDASeq (v2.12.0). Differential expression was determined, and PCA plots were prepared using DESeq (v1.18.1); differences in gene expression with a *P* value < 0.05 and a fold-change > 1.5 were considered significant differences. A correlation plot was prepared using ggplot2 (v2.2.1), and a heatmap was prepared using pheatmap (v1.0.10). Gene ontology analysis was performed using clusterProfiler (v3.6.0).

### Statistical analysis

In general, the results are presented as the mean ± SD (standard deviation) calculated using Microsoft Excel and GraphPad Prism from at least three biological repeats. For all experiments except the determination of survival, data were analyzed by Student’s *t*-tests, and differences were considered statistically significant if *p* < 0.05. The survival of the two groups was analyzed using a log-rank test, and differences were considered statistically significant if *p* < 0.05. *p < 0.05, **p < 0.01, ***p < 0.001.

## Supplementary information

Supplementary Information

Supplementary Information

## Data Availability

The datasets generated and/or analyzed during the current study are available from the corresponding author upon reasonable request.
