# Supplementary material for: METTL13 is essential for the survival of acute myeloid leukemia cells by regulating MYC
Source: Cell Death Discov. 2025 May 17;11:240. doi: 10.1038/s41420-025-02512-x (PMC12085568; doi:10.1038/s41420-025-02512-x)
Supplement: Supplementary file 1 — Supplementary Information [file 41420_2025_2512_MOESM1_ESM.pdf]

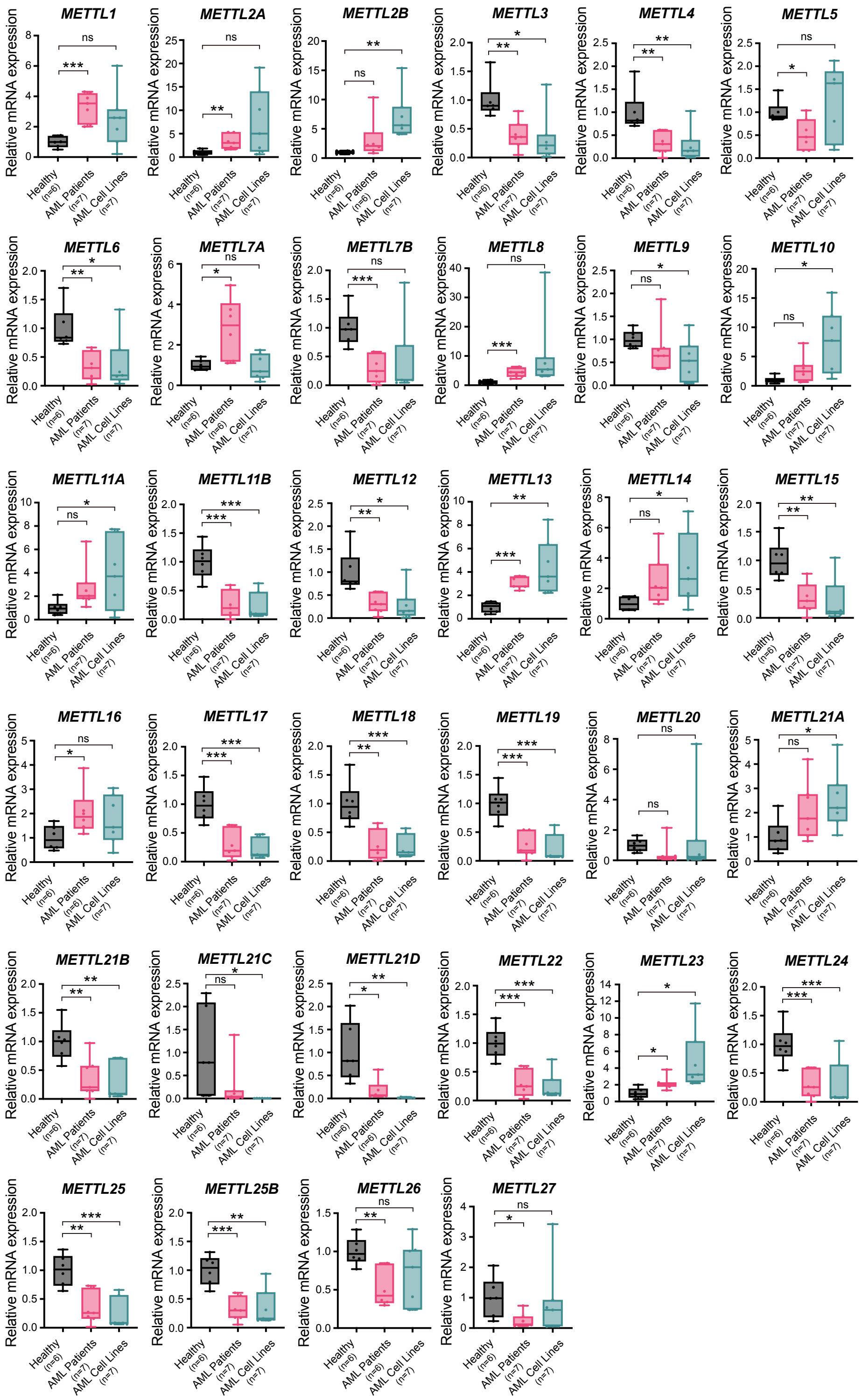

Fig. S1 Expression levels of METTL family in healthy individuals, AML samples and cell lines (HL-60, K562, U937, MOLM-13, MV-4-11, KG1a and Kasumi-1) by RT-qPCR. Data were presented as mean ± SD (Student's test, *$p < 0.05$, **$p < 0.01$, ***$p < 0.001$).

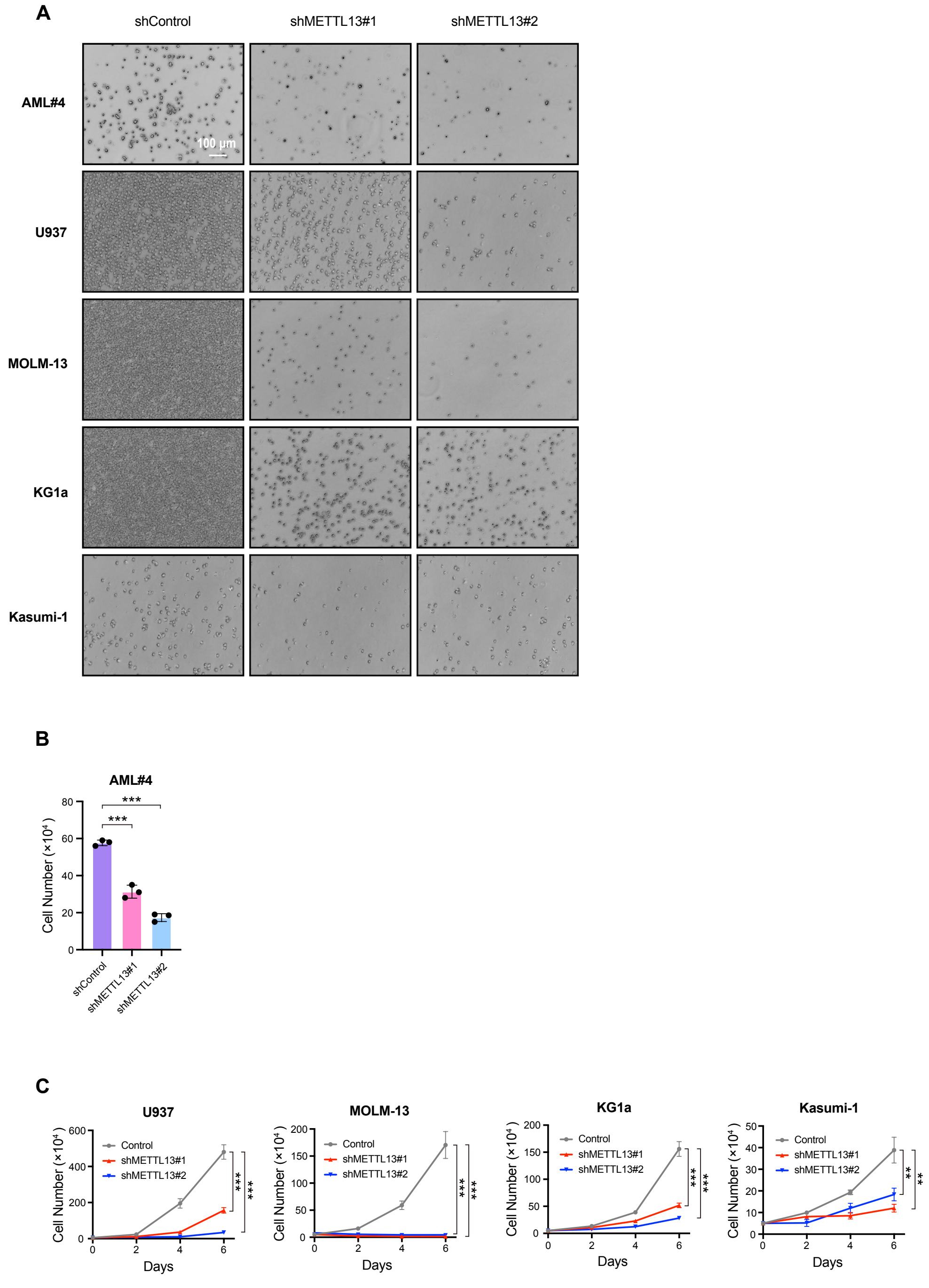

Fig. S2 METTL13 knockdown inhibited AML cell proliferation. (A) Representative growth images of an AML sample and cell lines at day 4 after METTL13 knockdown. **(B)** The cell counts of bone marrow mononuclear cells from an AML patient on day 4 after METTL13 knockdown. **(C)** Proliferation curves of AML cell lines after METTL13 knockdown. Data were presented as mean ± SD (Student's test, *p < 0.05, ***$p < 0.01$*, ****$p < 0.001$*).

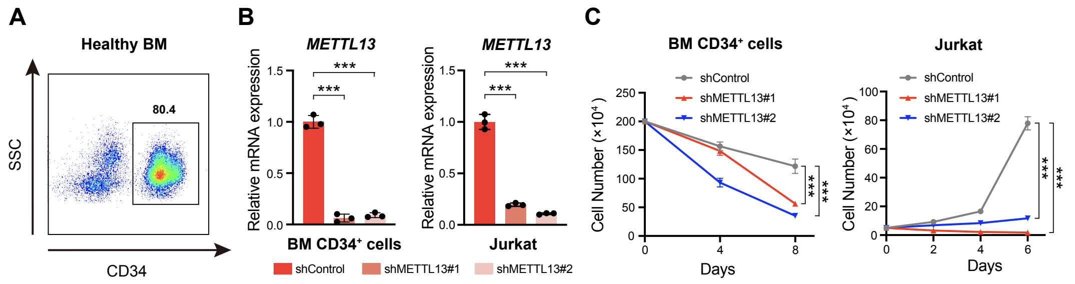

Fig. S3 METTL13 knockdown had modest inhibitory effects on normal hematopoietic cells and ALL cells. (A) The proportion of $CD34^+$ cells isolated from a healthy donor bone marrow via magnetic bead sorting was validated using flow cytometry. **(B)** Validation of the knockdown effect of METTL13 using RT-qPCR and Western blot in $CD34^+$ cells and Jurkat cells. **(C)** Proliferation trend of $CD34^+$ cells and Jurkat cells by cell counting after METTL13 knockdown. Data were presented as mean ± SD (Student's test, *p < 0.05, ***p < 0.01*, ****p < 0.001*).

Supplementary Table 1: RT-qPCR primers

**Primers**	**Sequence (5'→3')**
METTL1-F	GGCAACGTGCTCACTCCAA
METTL1-R	CACAGCCTATGTCTGCAAACT
METTL2A-F	ATGTGGAGTGGTCGGAAGAG
METTL2A-R	CCAGTATTTGTGGGCATTGATCT
METTL2B-F	GCCGCTATGACATGGCTCAGCT
METTL2B-R	AGTCGGCGGTCCACCAGATTCT
METTL3-F	CATTGCCCACTGATGCTGTG
METTL3-R	AGGCTTTCTACCCCATCTTGA
METTL4-F	TTGTCACCCCTGCAAATACAG
METTL4-R	TCCACAGACCAAGAGGGATAAAG
METTL5-F	AAGGAACTAGAGAGTCGCCTG
METTL5-R	GCGGCCTGGTAGGATACTG
METTL6-F	CAGGCAAGGATTCTCACCTCT
METTL6-R	TGGTCCAGTGTCTGTCTTTGA
METTL7A-F	CAGAGTGCTGAGACCGGGA
METTL7A-R	CTGGTCAGGTTGCACCCATC
METTL7B-F	CCTGCCTAGACCCAAATCCC
METTL7B-R	AAACCGCTCATATTGGAGGTG
METTL8-F	GGGATCACATGCAGTGGTCTA
METTL8-R	CCCTCAACAGCCAATTACGAT
METTL9-F	AATGGCAGAATACGGGGTTCC
METTL9-R	GCCTCTAGTTGGCTCCAAGAC
METTL10-F	TCAGTGCTTGATATTGGAACTGG
METTL10-R	GCTGAAGTGGAAAAGATCGCTTG
METTL11A-F	GCAGAGGTTTTTGAGGGAAGG
METTL11A-R	CCAGGAAGTCCTCCGTTATGTC
METTL11B-F	GAAGACCGACGATGAACTCTG
METTL11B-R	AGCAGGTAGAGATAGCTCTGAAA
METTL12-F	TCCCCACCTTCGACTGGTT
METTL12-R	GTGTGGAGATTTGGTGTAGAGG
METTL13-F	GGGATGACAAGCGATACTTCC
METTL13-R	GCCAGCGATCATGGCTTTG
METTL14-F	AGTGCCGACAGCATTGGTG
METTL14-R	GGAGCAGAGGTATCATAGGAAGC
METTL15-F	CATGTTGGTTGGAATCTGGCA
METTL15-R	TGAGTTTGATCTGTTTGCTCCC
METTL16-F	TTCTGTCAAGGTCGGACAATG
METTL16-R	CAGCACCACGAATGTTATGGG
METTL17-F	GCGGCACTGAAGTGTCTACTG

METTL17-R	GGTCACTCCGGGTACTAAGG
METTL18-F	TCAAGGAACTTGGAGCCACAT
METTL18-R	GCCTCCCTCATAAACACCTGTAA
METTL19-F	ACGGCATGTTGCGAACTTGA
METTL19-R	CAACTCCGAATTGCCACGG
METTL20-F	CCAAGCCCTGTCTAGGTATCT
METTL20-R	CTCCACATCCACTCCCAAGA
METTL21A-F	GAGGAGACCACGGAATTTGGG
METTL21A-R	CAGGTATGTGGAAAGAACGATGG
METTL21B-F	ATCCCGAATCTGAGTCGGAAT
METTL21B-R	ACTCTCGAAATAATTGCACAGGC
METTL21C-F	TCCTACAGATTACGCCAGCTAC
METTL21C-R	GTCCGGCACCAATTTCAAGT
METTL21D-F	TCACCGATCTTGAGGAATTGC
METTL21D-R	TATGCAGTCGGCCATCAGTAT
METTL22-F	ATGTTCACACAAAGGAGCCTC
METTL22-R	CACGTCCAAATCCCCATCCTC
METTL23-F	TTCTGGCTCTACCACCACAAG
METTL23-R	TGACCAGTCAGCACTCCTAAC
METTL24-F	CCAACAACGGATGTGAAGTGC
METTL24-R	GGTGATACCAAAGGTGCTGACT
METTL25-F	TGTAGAGCCTACTTCTTCACAGC
METTL25-R	GTCATGGAGTTCTGAATCAGCAG
METTL25B-F	CGTCAGGTACAGGTCAGTGTG
METTL25B-R	AATGGAGCTGTTAGTCGGGAG
METTL26-F	CCCTATGCCATCAATGGGAAG
METTL26-R	GAGCATCAGGTCAAAGTCCAC
METTL27-F	CCTGCAATGCGATACCTGAG
METTL27-R	CGCCTTCCACTTTCGGTACA
MYC-F	GGCTCCTGGCAAAAGGTCA
MYC-R	CTGCGTAGTTGTGCTGATGT
GAPDH-F	GGAGCGAGATCCCTCCAAAAT
GAPDH-R	GGCTGTTGTCATACTTCTCATGG

Supplementary Table 2: Antibodies

Antibodies	Vendor	Cat#	Dilution
Rabbit anti-METTL13	Bethyl	A304-195A	1:2000 for WB
Rabbit anti-MYC	CST	D84C12	1:2000 for WB
HRP GAPDH	KangChen	KC-5G5	1:4000 for WB
HRP Goat anti-Rabbit IgG	Proteintech	SA00001-2	1:2000 for WB
APC anti-CD11b	Biolegend	379906	1:100 for FACS
APC IgG2a, κ	Biolegend	402206	1:100 for FACS
DAPI	Sigma	D9542	1:100 for FACS

Supplementary Table 3: Evidence for METTL13 knockdown in RNA-seq

	HL-60	K562
**gene_id**	ENSG00000010165	ENSG00000010165
**gene_name**	METTL13	METTL13
**log2FoldChange**	-1.336111499	-1.701273315
**shControl-1**	2722	2328
**shControl-2**	2444	2502
**shMETTL13#1-1**	1065	866
**shMETTL13#1-2**	1140	854
**shMETTL13#2-1**	888	612
**shMETTL13#2-2**	1106	642
**baseMean**	1533.289478	1294.630508
**lfcSE**	0.144441372	0.204983765
**stat**	-9.250199444	-8.299551501
**pvalue**	2.24E-20	1.05E-16
**padj**	1.71E-17	2.64E-14
**significance**	Down	Down
