# Supplementary figures and images for: METTL13 is essential for the survival of acute myeloid leukemia cells by regulating MYC
Source: Cell Death Discov. 2025 May 17;11:240. doi: 10.1038/s41420-025-02512-x (PMC12085568; doi:10.1038/s41420-025-02512-x)

Original Western Blot

Fig. 1D

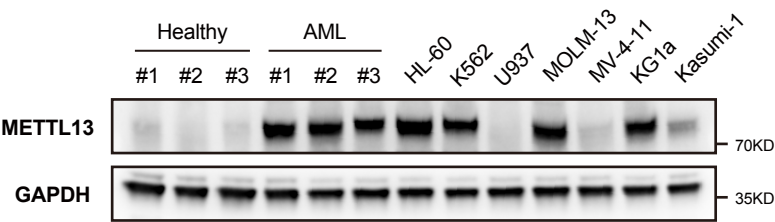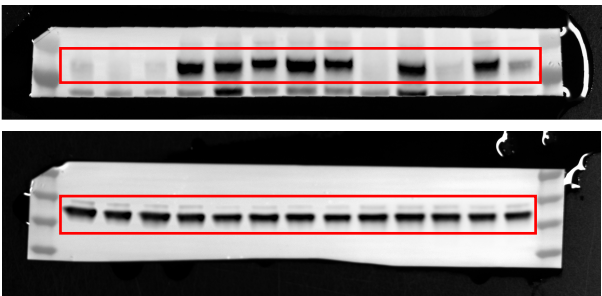

Fig. 2B

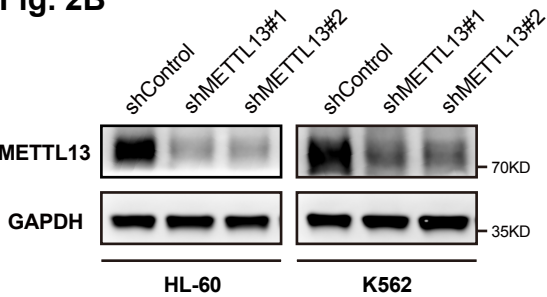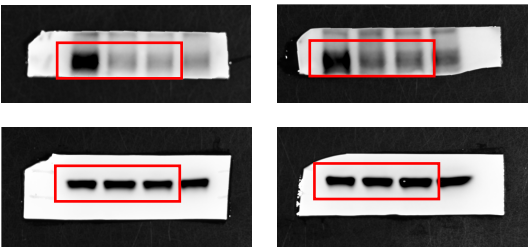

Fig. 3B

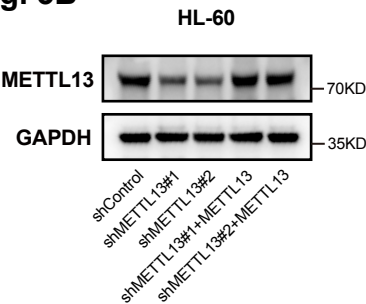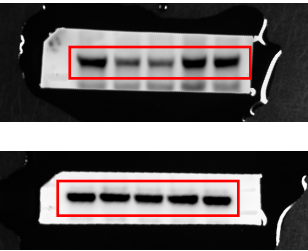

Fig. 5F

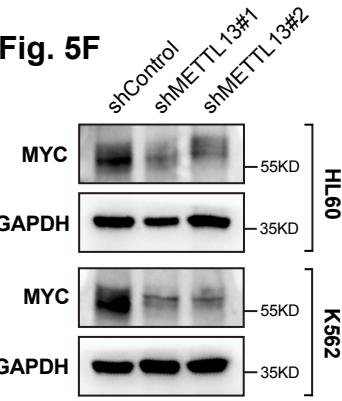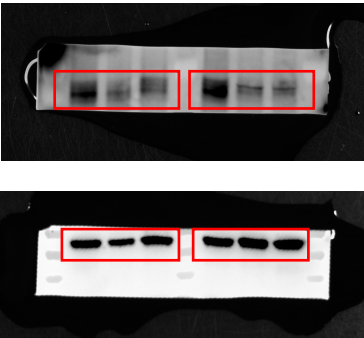

Fig. 6B

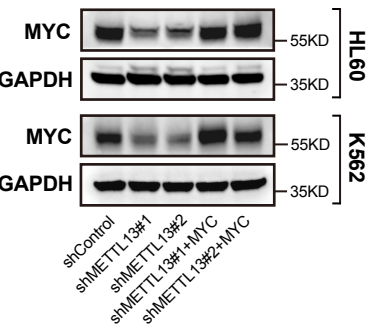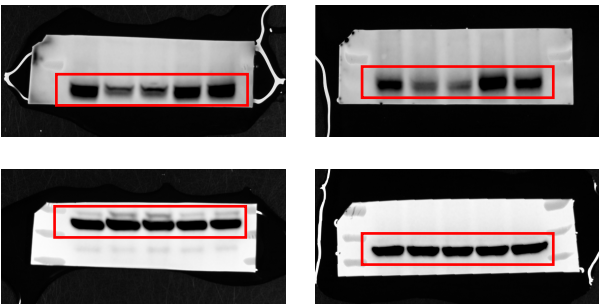

Supplement: Supplementary file 2 — Supplementary Information [file 41420_2025_2512_MOESM2_ESM.pdf]
